# Collagen-Inducing Compounds from Chihuahuan Desert Plants for Potential Skin Bioink 3D Printing Applications: A Narrative Review

**DOI:** 10.3390/jfb17020074

**Published:** 2026-02-02

**Authors:** Andrea I. Morales Cardona, René Gerardo Escobedo-Gonzalez, Alma Angelica Vazquez-Flores, Edgar Daniel Moyers-Montoya, Carlos Alberto Martinez Pérez

**Affiliations:** 1 Instituto de Ciencias Biomédicas, Universidad Autónoma de Ciudad Juárez, Henry Dunant #4600, Ciudad Juárez 32310, Mexico; moralesandrea6@gmail.com (A.I.M.C.); alma.vazquez@uacj.mx (A.A.V.-F.); 2 Department of Industrial Maintenance and Nanotechnology, Master’s Program in Sustainable Industrial Systems Engineering, Technological University of Juarez City, Ciudad Juarez 32695, Mexico; rene_escobedo@utcj.edu.mx; 3 Instituto de Ingeniería y Tecnología, Universidad Autónoma de Ciudad Juárez, Ave. Del Charro 450 Norte, Ciudad Juárez 32310, Mexico

**Keywords:** 3D Bioprinting, skin tissue engineering, collagen modulation, natural bioactives, phytochemistry, molecular docking, Chihuahua flora, regenerative medicine

## Abstract

This review synthetizes experimental evidence on collagen-related bioactivity and the biomaterial potential of plant species native to the Chihuahuan Desert, aiming to identify natural compounds that could enhance next-generation dermal bioinks for 3D bioprinting. A structured search across major databases included studies characterizing plant extracts or metabolites, with reported effects on collagen synthesis, fibroblast activity, inflammation, oxidative balance, or interactions with polymers commonly used in skin-engineering materials being developed. Evidence was organized thematically to reveal mechanistic patterns despite methodological heterogeneity. Several species, among them *Larrea tridentata*, *Opuntia* spp., *Aloe* spp., *Matricaria chamomilla*, *Simmondsia chinensis*, *Prosopis glandulosa*, and *Artemisia ludoviciana*, repeatedly demonstrated the presence of bioactive metabolites such as lignans, flavonoids, phenolic acids, terpenoids, and polysaccharides. These compounds support pathways central to extracellular matrix repair, including stimulation of fibroblast migration and collagen I/III expression, modulation of inflammatory cascades, antioxidant protection, and stabilization of ECM structures. Notably, several metabolites also influence viscoelastic and crosslinking behaviors, suggesting that they may enhance the printability, mechanical stability, and cell-supportive properties of collagen-, GelMA-, and hyaluronic acid-based bioinks. The review also reflects on the bioethical and sustainability considerations regarding endemic floral resources, highlighting the importance of responsible sourcing, conservation extraction practices, and alignment with international biodiversity and access to benefit/sharing frameworks. Taken together, these findings point to a promising, yet largely unexplored, opportunity: integrating regionally derived phytochemicals into bioinks to create biologically active, environmentally conscious, and clinically relevant materials capable of improving collagen remodeling and regenerative outcomes in 3D-printed skin.

## 1. Introduction

In recent years, three-dimensional bioprinting (3D bioprinting) has become a key tool in regenerative medicine. This technology makes it possible to fabricate skin-like structures with a level of detail that was previously difficult to achieve, and this is especially valuable when attempting to reproduce the natural architecture of human skin. Although several challenges remain, one of the most significant issues involves the development of bioinks that not only preserve shape and provide the mechanical stability required but also deliver biological signals that support tissue regeneration. Processes such as collagen stimulation, fibroblast proliferation, and proper extracellular matrix (ECM) organization are essential for effective tissue repair [1,2].

**Figure 1:**
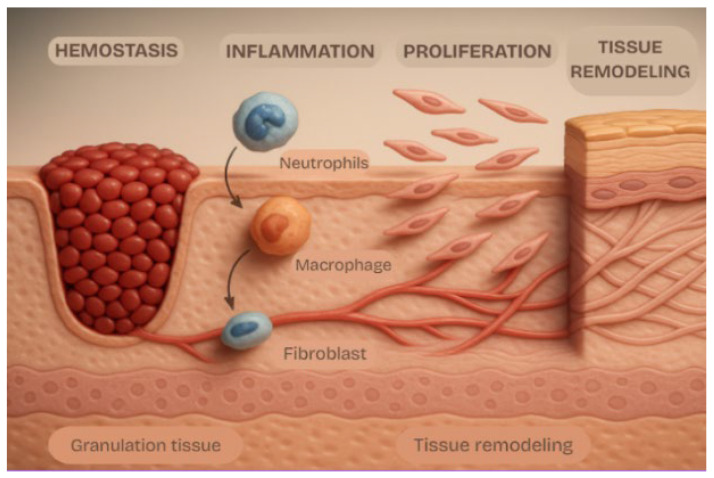
Steps of wound healing: hemostasis, inflammation, proliferation, and tissue remodeling. Note: Image generated with Chat GPT-5.1 on 20 November 2025. The prompt used was as follows: “representation of the steps in wound healing in a 3D model.”.

This need becomes even clearer when considering the clinical burden associated with skin injuries (Figure 1). The skin—by far the largest and one of the most complex organs in the human body—plays protective, immunological, thermoregulatory, and sensory roles that sustain homeostasis [3]. Its extracellular matrix, primarily composed of type I and type III collagen, provides the tensile strength and elasticity characteristic of healthy tissue, while its vascular and cellular networks coordinate the body’s repair response [4]. Moreover, the skin contains a complex network of vasculatures, neurons, immune cells, hair follicles, sebaceous glands, and sweat glands that coordinate systemic communication and repair [4,5].

However, severe injuries such as extensive burns, chronic ulcers, or deep trauma can surpass the skin’s natural regenerative capacity, leading to slow or incomplete healing processes that significantly impact patient quality of life. Epidemiological evidence indicates that more than 11 million people require medical attention for burns annually, while approximately 6.5 million individuals suffer from chronic wounds, making this an underrecognized “silent epidemic” that disproportionately affects older adults and individuals with comorbidities such as obesity and diabetes [6,7]. Superficial wounds may regenerate spontaneously, yet full-thickness injuries generally require clinical intervention and often result in incomplete closure, infection, fibrosis, or delayed healing [8,9]

Three-dimensional bioprinting has emerged as one of the most promising approaches to overcome these limitations, mainly because it allows the fabrication of multilayered, cell-laden structures that replicate the composition of the extracellular matrix and support early neovascularization. While natural polymers such as alginate, gelatin, chitosan, fibrin, and hyaluronic acid are widely used due to their biocompatibility, they often lack sufficient mechanical strength, long-term stability, vascularization potential, or the capacity to regenerate complex cutaneous appendages [1,2]. Consequently, there is growing interest in biofunctional additives—including decellularized extracellular matrix (dECM) and plant-derived biomaterials—to improve the biological performance of bioinks. Plant-based compounds such as polysaccharides, gums, proteins, essential oils, and secondary metabolites have demonstrated biodegradability, biocompatibility, and relevant bioactivity, positioning them as attractive candidates to enhance scaffold performance [10,11].

Mexico, known as one of the most biodiverse countries in the world, offers an exceptionally rich reservoir of botanically derived biomaterials, and this point gains even more relevance when considering that the state of Chihuahua alone contains over 20% of the nation’s medicinal flora [12]. Species such as *Aloe vera*, *Larrea tridentata*, *Matricaria chamomilla*, and *Simmondsia chinensis* have demonstrated potential to promote collagen synthesis and support skin regeneration. Their phytochemical profiles include phenolic compounds, terpenoids, and alkaloids that can act on pathways central to dermal repair. These include NF-κB, Nrf2, PI3K/Akt, and arachidonic acid metabolism, regulating inflammation, oxidative stress, fibroblast proliferation, and ECM remodeling [13,14]. Other natural resources with demonstrable effects on health are *Opuntia phaceacata*, *Propsis grandulosa*, *Artemisa ludoviciana*, *Dasylirion wheeleri*.

**Figure 2:**
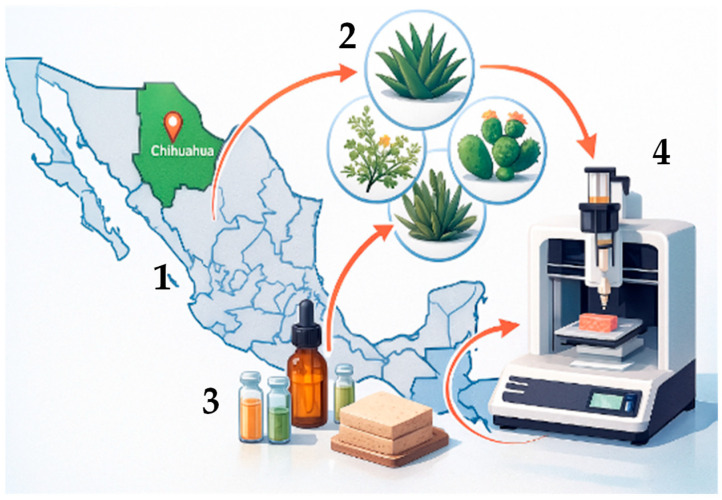
Natural plant-derived metabolites from Chihuahuan flora support collagen-related pathways for skin regeneration and show potential for incorporation into bioinks. Note: Image generated with Chat GPT-5.1 on 20 November 2025. The prompt used was as follows: “Diagram illustrating a three-stage flow process: (1) A map of Mexico with the state of Chihuahua highlighted in green. (2) A circle showing a collage of common desert plants from Chihuahua (such as *agave*, *prickly pear*, and *creosote bush*/*Gobernadora*). (3) An arrow connecting the plants to vials and bottles representing metabolite extraction. (4) An arrow connecting the vials to a 3D bioprinter that is extruding into a tissue-like material. The style should be a clean scientific illustration.”.

Plants native to the Chihuahuan Desert are particularly noteworthy because their adaptive ability to extreme environmental stressors such as intense ultraviolet radiation, severe aridity, and abrupt temperature fluctuations promotes the accumulation of potent secondary metabolites with antioxidant and regenerative activity [12]. However, despite this high biochemical richness, the literature remains fragmented. Existing studies often examine isolated compounds or specific plant extracts without integrating their collagen-inducing effects or evaluating their translational relevance for next-generation skin bioinks. To provide a quick visual overview, Figure 2 presents a graphical summary that shows a close-up of one of the representative plants. It is an AI-generated image, and while such tools can sometimes introduce small visual inaccuracies, the main purpose of the figure is simply to provide a general visual context about the type of flora we refer to throughout the manuscript. Rather than serving as a strict botanical reference, the figure works as a conceptual summary that helps illustrate why these plants are relevant when discussing collagen stimulation and dermal regeneration.

To address this gap, the present work involves a comprehensive search to identify, characterize, and integrate evidence on compounds which induced natural collagen production and wound healing derived from chemical compounds obtained from Chihuahuan Desert plants. We examine their major phytochemical classes, summarize their interactions with key dermal signaling pathways, and evaluate their potential incorporation as biofunctional additives in bioinks for 3D bioprinting. This framework seeks to support the rational development of biologically active bioinks capable of enhancing dermal regeneration and minimizing fibrosis in engineered skin constructs.

Although many of the studies included in this review do not specify whether their plant material originated directly from the state of Chihuahua, the taxa examined are consistently recognized in floristic, ethnopharmacological, and ecological records as integral components of the medicinal flora naturally distributed across the Chihuahuan Desert. This broader biogeographical perspective allows us to bridge global experimental evidence with the regional botanical landscape, acknowledging that the biological activities reported in the literature pertain to species that also inhabit—and in many cases thrive within—the environmental conditions of Northern Mexico. Our intention is not to attribute specific biochemical outcomes to a localized provenance, but rather to illuminate the strategic potential embedded in Chihuahua’s native and naturalized plant resources.

By contextualizing dispersed experimental findings within this regional framework, we underscore a deeper message: the flora of the Chihuahuan Desert represents a biologically rich and relatively underexplored source of phytochemical diversity that could inform the next generation of regenerative biomaterials. These species, shaped by extreme desert conditions, frequently accumulate metabolites with structural, antioxidant, and anti-inflammatory properties that align with the functional demands of collagen-supportive hydrogels and 3D dermal bioinks. Highlighting their presence in Chihuahua draws attention to a unique opportunity for sustainable territory-based innovation that integrates local biodiversity, responsible bioprospecting, and translational biomaterials research.

Hence, this review positions regional flora not merely as passive elements of biodiversity but as a promising foundation for developing biologically active, environmentally grounded, and clinically relevant materials for skin regeneration. This perspective strengthens the scientific relevance of the region’s botanical heritage and reinforces its potential to contribute meaningfully to emerging fields such as 3D bioprinting and advanced dermal engineering.

## 2. Methods

This review followed a structured literature mapping approach designed to identify and organize experimental evidence related to collagen-associated biological activity and biomaterial relevance of plant species native to the Chihuahuan Desert. A broad and structured search was conducted across PubMed, Scopus, Web of Science, and Google Scholar from January 2020 up to October 2025 using combinations of botanical terms, collagen with related keywords, fibroblast and extracellular matrix descriptors, and terminology associated with natural polymers and hydrogel systems. Additional studies were located through manual screening of reference lists from relevant publications. All retrieved records were compiled in a reference manager, and the duplicates were removed prior to screening [15].

Title and abstract screening, followed by full text assessment, was performed independently by two reviewers according to predefined criteria. Studies were included when they achieved the following: (a) examined native or naturalized plants in the Chihuahuan region; (b) characterized extracts or isolated metabolites; and (c) reported experimental outcomes related to collagen synthesis, fibroblast activity, oxidative balance, inflammatory modulation, or interactions with biopolymers commonly used in dermal scaffolds. Exclusion criteria removed papers lacking phytochemical identification, studies unrelated to dermal or ECM biology, narrative articles, or publications with insufficient methodological detail. Disagreements in study selection were resolved through discussion [16].

For each eligible study, data extraction followed a standardized template capturing botanical identity, extraction procedures, principal metabolite classes, reported biochemical mechanisms, dermal endpoints, and any described interactions with collagen, gelatin, hyaluronic acid, alginate, or other natural polymers [17]. Given the heterogeneity of experimental designs and outcome measures, no quantitative pooling was attempted. Instead, extracted information was organized thematically to identify recurring mechanistic patterns, outline the metabolites form Chihuahuan sources with conceptual relevance for dermal bio fabrication, and highlight knowledge gaps requiring future experimental validation within bioink formulations.

## 3. Results

### 3.1. Chronological Development of Phytochemical Research in Chihuahuan Desert Flora

Understanding the biochemical potential of Chihuahuan Desert plants really requires looking at the problem from a chronological angle, because the identification and characterization of key bioactive compounds has unfolded progressively over the last decade. Table 1 brings together the most relevant findings related to the antioxidant, anti-inflammatory, and matrix-modulating properties of regional species, and it highlights those compounds that may be particularly valuable for dermal regeneration and collagen-support applications.

This timeline blends both the discovery of phytochemical constituents and the early biological evidence supporting their activity, placing each study within its scientific and geographical context. Although not all investigations evaluated fibroblast-related or dermal outcomes directly, several foundational works—especially those centered on phenolic composition and antioxidant mechanisms—offer essential biochemical justification for considering these plants within biomaterial and bioink research.

The results of the search strategy allowed us to develop Table 1, which highlights the wound healing properties of endemic plants from Chihuahua and their use in some materials. It is also important to point out that Table 1 brings together several key contributions from different research groups in Northern Mexico, including the work of De la Rosa and Álvarez-Parrilla, whose characterization of phenolics from pecan shells and kernels has served as a solid biochemical base for understanding antioxidant capacity in regional plant materials. Although there are still no studies that directly evaluate their properties in skin-level wound healing, the characterization of their antioxidant compounds makes this type of research is especially promising and opens the door to deeper exploration in dermal regeneration.

Later studies on *Larrea tridentata*, *Opuntia* spp., *Prosopis glandulosa*, *Artemisia ludoviciana*, different *Agave* species, and others show how evidence has gradually strengthened toward dermal-relevant bioactivity, eventually leading to more recent efforts exploring how these compounds might be integrated into hydrogels and bioinks. Much of this progress builds on decades of groundwork dedicated to the medicinal flora of Chihuahua, especially the extensive work of Royo-Márquez et al. [12]. Their detailed identification, compilation, and collaborative documentation of medicinal plants from the region have been essential for understanding what these species can offer in terms of health applications. Thanks to that early and thorough mapping of local botanical resources, later biochemical, pharmacological, and biomaterials-focused studies were able to move forward with clearer direction, building on a foundation that continues to guide exploration of the regenerative and therapeutic potential of the Chihuahuan Desert today.

#### Molecular Representation of Key Bioactive Compounds

**Figure 3:**
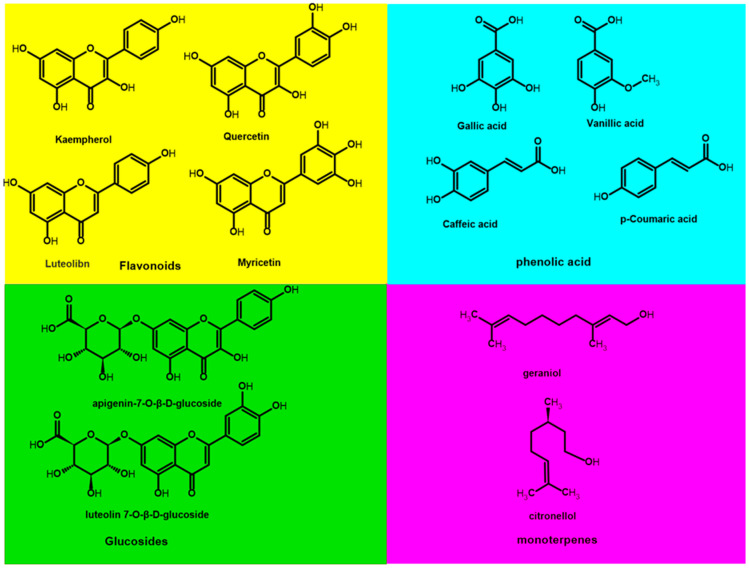
The molecular structure of selected key bioactive compounds reported by research groups in Table 1.

The following figure (Figure 3) shows the molecular structure of selected key bioactive compounds, grouped into four families: phenolic acids, flavonoids, glycosides, and monoterpenes, present in most plant specimens. Among the molecules are flavonoids such as kaempferol, quercetin, luteolibin, and myricetin, while phenolic acids include gallic acids and caffeic acid, among others. The glycoside molecule includes apigenin-7-O-β-D-glucoside. Other important molecules include nordihydroguaiaretic acid, β-sitosterol, phosphatidylcholine, sphingolipids, squalene, quercetin, lutein, α-tocotrienol, gallic acid, ellagic acid, and acemannan. Each family is displayed in an individual panel for visual comparison, highlighting the chemical diversity typical of medicinal plant species from arid regions.

### 3.2. Biopolymers and Plant-Derived Wound-Healing Agents

According to the studies reviewed, eight major natural biopolymers—hyaluronic acid, starch, cellulose, chitosan, alginate, gelatin, and pectin—emerged as the most recurrent scaffolding materials in wound-healing formulations. These biomaterials are widely recognized for their ability to maintain a moist microenvironment, support oxygen permeability, and stimulate cellular proliferation, thereby fostering conditions conducive to accelerated tissue repair. Both clinical and pre-clinical investigations consistently validate their biocompatibility and functional performance across a wide range of regenerative applications [41]. Importantly, these polymers also provide a robust platform for incorporating bioactive molecules aimed at enhancing collagen synthesis—a central focus of this review—as they can serve as carriers, stabilizers, or synergistic scaffolds for phytochemical compounds with pro-collagen activity [42].

Building upon this foundation, the successful translation of Chihuahuan flora-derived phytochemicals into functional dermal bioinks requires a precise understanding of how these natural metabolites interact with the biopolymers. Their physicochemical compatibility with commonly used biomaterial platforms, such as gelatin methacrylate (GelMA), collagen, alginate, and hyaluronic acid (HA), is essential not only for ensuring printability, structural stability, and biological relevance, but also for enabling the controlled release and mechanistic activation of collagen-stimulating pathways. This integration step directly aligns with the primary objective of the article: to identify and describe novel strategies for enhancing collagen production using naturally occurring compounds from the Chihuahuan region. By mapping how these phytochemicals can be incorporated into established bioink matrices, the review advances a pathway toward innovative dermal regeneration approaches that merge regional biodiversity with modern bio fabrication technologies.

These base matrices are widely used in biomaterials and bioprinting and exhibit crosslinking pathways and rheological profiles that can be markedly tuned by natural additives such as phenolic acids and terpenoids. For instance, adding *Larrea tridentata* extracts enriched in nordihydroguaiaretic acid (NDGA) to GelMA formulations could improve thermal stability and oxidative resistance, owing to NDGA’s strong antioxidant activity [43]. Likewise, ellagic acid derivatives from *Opuntia* species may function as supplementary crosslinkers that enhance print fidelity. Although ellagic acid is often treated as a model analog of gallic acid, it is gallic acid and its derivatives that are more frequently employed as crosslinking agents for proteins like gelatin, strengthening the bioink’s mechanical properties and enabling high-resolution bioprinting [43,44,45].

From a processing standpoint, the compatibility of these compounds with photo-initiators such as Irgacure 2959 or LAP (lithium phenyl-2,4,6-trimethylbenzoylphosphinate) should be assessed to ensure consistent polymerization kinetics and avoid free-radical inhibition. Additionally, their solubility and thermal degradation profiles must align with typical bio fabrication conditions (25–37 °C for extrusion printing) [46].

Regarding regulatory considerations, the inclusion of phytochemicals of botanical origin in medical bioinks would require toxicological validation following FDA 21 CFR Part 73 and EMA guidelines on herbal-derived excipients. Therefore, establishing biocompatibility, batch-to-batch reproducibility, and compliance with Good Manufacturing Practices (GMPs) are essential steps before clinical translation [47].

Overall, mapping the interplay between plant-derived bioactive compounds and conventional dermal bioinks provides a robust scientific basis for engineering hybrid formulations that unite biological activity with the structural performance required for advanced 3D bioprinting. Across the evaluated studies, certain botanical resources—particularly *Larrea tridentata*, *Matricaria chamomilla* L., *Simmondsia chinensis*, and *Opuntia* spp.—consistently emerged as the most effective in supporting tissue regeneration and promoting accelerated skin repair. Their phytochemical profiles activate well-characterized molecular pathways involved in endogenous collagen synthesis, fibroblast proliferation, antioxidant protection, and extracellular matrix remodeling. These mechanisms collectively underscore the relevance of these species as promising candidates for integration into next-generation dermal bioinks, setting the stage for the detailed compound-specific analyses presented in the following sections.

#### 3.2.1. *Larrea tridentata* (*Chaparral*)

**Figure 4:**
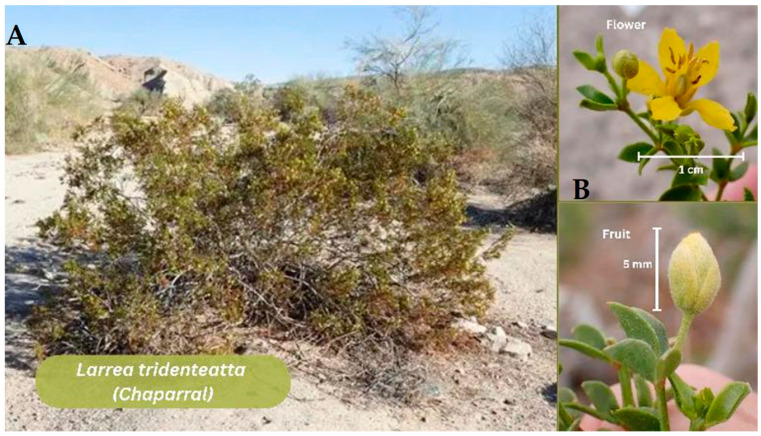
*Larrea tridentata (creosote bush).* (**A**) Mature *Larrea tridentata* shrub in its desert habitat. (**B**) Close-up of the characteristic yellow flower (~1 cm). Developing fruit (~5 mm) showing the typical globose morphology. Note: Figure created in Canva (Canva Visual Suite 2.0) using images from Adobe Stock. Photo rights © are the Author’s.

Plant Extract-Enriched biomaterials: The extracts of this plant (Figure 4) have been incorporated into hydrogels as previously noted in Table 1. In a complementary work, Tovar-Carrillo and colleagues prepare cellulose hydrogels with 1–5% of LT extract, which increased swelling capacity. Also, hydrogel films were applied intramuscularly into Wistar rats, to analyze their cytocompatibility and biocompatibility, enhanced tissue integration, and produced no toxicity or chronic inflammation over 90 days [48].

It is convenient to note that some natural products obtained from this species have relevant activities and have been used in biomaterials design. In this sense, the following paragraphs were focused on these compounds and their applications.

Nordihydroguaiaretic acid (NDGA) Topical Application (Skin Model): In a TPA-induced skin inflammation model in female Swiss albino mice, NDGA significantly reduced myeloperoxidase (MPO) activity, skin edema, and epidermal thickening. It lowered lipid peroxidation (LPO), hydrogen peroxide (H_2_O_2_), and xanthine oxidase (XO) activity while restoring antioxidant defenses (GSH, CAT, SOD, GPx, GR) [49].

Ellagic Acid (EA) in Corrosive Esophageal Burn (CEB) Model: In a 24-rat CEB model induced with 20% NaOH, oral EA (30 mg/kg daily, 28 days) significantly reduced malondialdehyde (MDA) levels, improved histopathological scores (reduced fibrosis, necrosis, and inflammation), and lowered the stenosis index. Immunohistochemistry revealed upregulation of epidermal growth factor (EGF), suggesting EA promotes mucosal regeneration via EGF signaling [50].

Ellagic Acid anti-inflammatory and antimicrobial effect in dermal fibroblast: A single in vitro study investigated the effects of ellagic acid and punicalagin on fibroblast activity and inflammatory markers under simulated chronic wound conditions. Human dermal fibroblasts were exposed to pro-inflammatory cytokines (IL-1β, IL-6, TNF-α) or bacterial lipopolysaccharides (LPSs) and subsequently treated with ellagic acid or punicalagin at concentrations of 10^−6^ M and 10^−7^ M. Both ellagic acid and punicalagin significantly enhanced fibroblast viability and migration in an inflammatory environment and decreased IL-1β and IL-6 secretion compared with untreated controls. Neither compound demonstrated measurable antimicrobial activity against the tested microorganisms at the concentrations used. The evidence suggests that ellagic acid and punicalagin may support wound healing by modulating inflammatory responses and enhancing fibroblast function. However, they lack direct antimicrobial effects. No in vivo data was available, and further animal or clinical studies are required to confirm therapeutic potential and clinical applicability [51].

Combined Interpretation: Evidence from NDGA, LT extract, and EA indicate robust antioxidant activity, inflammation suppression, restoration of endogenous antioxidant enzyme systems, and promotion of tissue repair. Despite their lack of direct antimicrobial activity, ellagic acid and punicalagin may support wound repair by modulating inflammation pathways and enhancing fibroblast function. Further in vivo and clinical studies are warranted to optimize dosing, confirm therapeutic relevance, and develop advanced biomaterial-based formulations incorporating these bioactive compounds.

#### 3.2.2. *Aloe vera*

**Figure 5:**
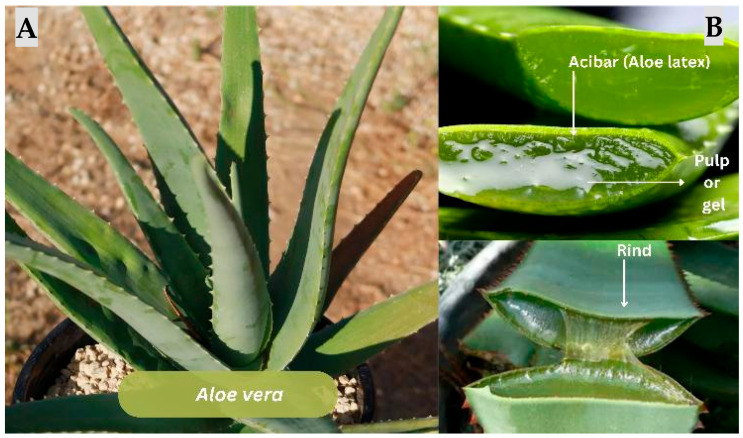
*Aloe vera*. Morphology and components. (**A**) *Aloe vera* plant displaying its thick, fleshy, lanceolate leaves arranged in a basal rosette. (**B**) Leaf cross-section indicating the three primary components: the outer rind (protective epidermal and cortical tissues), the acíbar (yellow aloe latex) located just beneath the rind, and the clear parenchymatous gel (inner mucilaginous pulp). Note: Figure created in Canva (Canva Visual Suite 2.0) using images from Adobe Stock. Photo rights © are the Author’s.

Based Hydrogels and Bioprinted Constructs: *Aloe vera* (Figure 5) hydrogels demonstrated multiple wound-healing benefits attributed to their bioactive compounds. Aloe-based hydrogels are three-dimensional, porous, and absorb exudate while permitting oxygen diffusion, which is essential for tissue repair [52,53,54]. Also, the *Aloe* compounds exhibit anti-inflammatory, antimicrobial, and antioxidant effects, collectively accelerating wound healing [52,55,56].

Regarding to therapeutic outcomes, it is convenient to highlight that ***Aloe*** formulations promoted faster epithelialization, encouraged fibroblast migration and collagen synthesis, reduced scarring, and provided superior pain relief compared to silver sulfadiazine [52,57]. Therefore, a commercial Aloe hydrogel with 1,2-propanediol and triethanolamine reduced total healing time by 29%, achieving full wound closure within 15 days in female Wistar rats. Additionally, another review says composite hydrogels with 10% (*w*/*v*) Aloe vera showed superior strength, elasticity, and absorption capacity [52,58,59].

Three-dimensionally Printed Hydrogels: Alginate/gelatin scaffolds with Aloe extract displayed optimal pore size (163.66 ± 14.45 μm) and tensile strength (16.39 ± 0.98 MPa), supported NHDF cell proliferation, and reduced inflammation in vivo. Bioprinted Aloe bioinks improved viscoelasticity and maintained stem cell viability [60].

Biological Activities of *Aloe vera* Phenolic and Polysaccharide Extracts: A study evaluated the biological activities of phenolic, and polysaccharide extracts from *Aloe vera* gel, obtained using ultrasound-assisted extraction (UAE). The phenolic extract contains compounds like aloin, ferulic acid, caffeic acid, and quercetin dihydrate. Meanwhile, the polysaccharide extracts are predominantly composed of mannose and glucose. At 0.25 mg/mL, the phenolic extract significantly inhibited pro-inflammatory cytokines: TNF-α decreased by 28%, and IL-8 by 11%.

At the same concentration, polysaccharide extracts reduced TNF-α by 17% and IL-8 by 8%. Both extracts showed a pro-inflammatory effect at 0.5 mg/mL, highlighting concentration-dependent activity. Also, the phenolic extract enhanced L929 fibroblast proliferation by 18% at 0.5 mg/mL and increased cell migration by 20%. The polysaccharide extract had comparatively lower effects on proliferation and migration. Meanwhile, the polysaccharide extract was more effective in promoting collagen synthesis, with a 25% increase. The phenolic extract increased collagen type I synthesis by 18%, indicating differential effects on ECM remodeling.

The phenolic extract showed superior antioxidant activity DPPH radical scavenging IC50: 2.50 mg/mL. ABTS assay: 16.47 mM TE/g. Inhibited intracellular reactive oxygen species (ROS) production by 46% at 0.5 mg/mL. The polysaccharide extract displayed comparatively lower antioxidant activity. The polysaccharide extract was effective against Staphylococcus aureus, showing a bacteriostatic effect at 25 mg/mL and a bactericidal effect at 50 mg/mL. The phenolic extract had negligible antibacterial effects [61]. In summary, phenolic extract is most effective during the initial inflammatory and proliferative stages due to its strong anti-inflammatory and antioxidant properties. The polysaccharide extract is more suitable for the remodeling stage, as it enhances collagen synthesis and provides antibacterial protection. The study suggests a potential synergistic use of these extracts within biomaterial-based dressings for accelerated chronic wound healing.

*Aloe saponaria*–Derived Extracellular Vesicles (AS-EVs) in Chronic Wound Healing. A study investigated the therapeutic potential of extracellular vesicles (EVs) isolated from *Aloe saponaria* peels using a polyethylene glycol (PEG)-based precipitation method. AS-EVs were tested on key cell types relevant to wound healing: RAW264.7 macrophages (anti-inflammatory effects), human dermal fibroblasts (HDFs, proliferation/migration), and human umbilical vein endothelial cells (HUVECs, angiogenesis). AS-EVs demonstrated no significant cytotoxicity across all tested concentrations. Fluorescent labeling confirmed successful uptake by macrophages, fibroblasts, and endothelial cells.

In lipopolysaccharide-stimulated RAW264.7 macrophages, AS-EV treatment significantly reduced expression of pro-inflammatory genes, including interleukin-6 (IL-6) and interleukin-1β (IL-1β), indicating attenuation of inflammatory signaling pathways.

AS-EVs promote proliferation and migration of HDFs, essential for granulation of tissue formation and re-epithelialization. Tube formation assays with HUVECs revealed enhanced angiogenesis, which is a critical step for nutrient supply and wound closure. Summary of Findings: AS-EVs were biocompatible, anti-inflammatory, and pro-regenerative. Their combined ability to suppress excessive inflammation, stimulate fibroblast activity, and promote angiogenesis suggests that AS-EVs may represent a promising cell-free therapeutic for chronic wound healing [62].

#### 3.2.3. *Matricaria chamomilla* (*Chamomile*)

**Figure 6:**
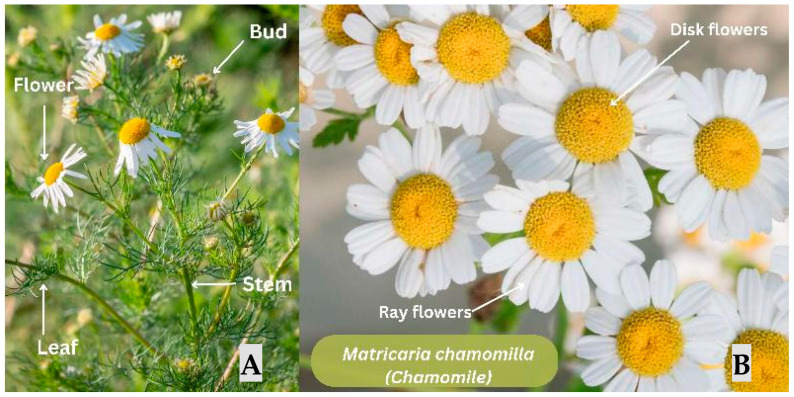
*Matricaria chamomilla* (Chamomile). (**A**) *M. chamomilla*, showing its slender stems, finely dissected leaves, and developing buds. (**B**) Detail of the capitulum highlighting ray flowers (white ligulate florets) and disk flowers (yellow tubular florets). Note: Figure created in Canva (Canva Visual Suite 2.0) using images from Adobe Stock. Photo rights © are the Author’s.

Several experimental studies evaluating the wound-healing potential of *Matricaria chamomilla* (Figure 6) were included. These studies investigated MC in different models: cutaneous wounds in healthy rats, diabetic wounds in rats, in vitro hydrogel development, and oral wounds in rats.

Cutaneous Wound Model in Healthy Rats: A study used 30 male albino rats with paired 5 mm full-thickness dorsal wounds. The left-side wounds were treated daily with MC essential oil, while right-side wounds served as untreated controls. Histological assessments were conducted at 3, 5, and 7 days. Healthy rat cutaneous wounds treated with MC showed accelerated contraction, with smaller wound areas by day 7. MC also accelerated epithelialization in cutaneous wounds, with complete epithelial coverage, hair follicle formation, and collagen remodeling by day 3 [63].

Diabetic Wound Model in Rats: This study included 48 male Wistar rats with streptozotocin-induced diabetes, randomized to receive MC 5% gel, MC 10% gel, gel-base vehicle, or no treatment. A 1 cm^2^ excisional wound was created and treated daily for 15 days. Stereological methods assessed wound closure, fibroblast proliferation, and vascular parameters. As a result, it was found that diabetic rats treated with MC 5% and 10% gels exhibited higher closure rates (5.96%/day and 6.14%/day, respectively) compared with controls (3.61%/day), demonstrating efficacy under impaired healing conditions and showed 99–106% higher fibroblast density than controls, indicating enhanced granulation and exhibited increased vessel length density and mean diameter, indicating enhanced re-vascularization [64].

Oral Wound Model in Rats: This study investigated 36 male Wistar rats with standardized 5 mm tongue wounds; the rats were treated twice daily with 0.02 mL of 10% MC ointment or left untreated. Animals were sacrificed at 3, 7, and 10 days. Outcomes included semi-quantitative inflammation scoring, fibroblast count, wound size, re-epithelialization, and collagen fiber percentage. Oral wound healing did not show significant differences in inflammation scores, suggesting tissue-specific effects of MC on inflammation. In oral wounds, re-epithelialization was greater at day 10 in MC-treated animals (0.1696 ± 0.0418 mm^2^ vs. 0.1309 ± 0.0497 mm^2^; *p* = 0.008), and collagen fiber percentage increased (8.26 ± 2.99% vs. 6.03 ± 3.18%; *p* = 0.022). Oral wounds showed no significant difference in fibroblast counts but improved alignment and granulation of tissue maturation [65].

Hydrogel Development Study: An in vitro study evaluated the effect of MC extract, starch solution, and photo-initiator concentration. UV-photopolymerized acrylic hydrogel properties, including swelling, tensile strength, and elasticity, optimize their potential as wound dressings. MC incorporation improved swelling, tensile strength, and elasticity [66].

Electrospun Multilayer Nanofibrous Patches: One study developed multilayer patches composed of a hydrophilic *chamomile*-loaded CECS/PVA nanofibrous layer (wound contact), a hydrophobic PCL nanofibrous layer (mechanical support), and a hybrid PCL/*chamomile*/CECS/PVA layer (interlayer cohesion). Chitosan was modified to CECS via Michael reaction and confirmed by ^1^H NMR and FTIR. Hydrogel and electrospun patch studies demonstrated potential for sustained local delivery of MC, supporting tissue remodeling through antioxidant and antibacterial effects [66,67] and SEM revealed continuous, smooth, bead-free fibers. Tensile strength ranged from 8.2 to 16.03 MPa. Antioxidant activity (6.6–38.0%) and antibacterial efficiency increased with higher MC content. *Chamomile* release was followed by a Fickian diffusion mechanism. MTT assays indicated acceptable cell viability for all mats, except 30 wt% MC-loaded mats, which reduced cell viability slightly [67].

Across studies, *Matricaria chamomilla* consistently promoted wound healing through multiple mechanisms: accelerated epithelialization, improved collagen deposition, enhanced fibroblast activity and alignment, and increased vascularization. Beneficial effects were observed in normal and impaired healing conditions, including diabetic wounds. Advanced delivery systems, such as hydrogels and electrospun multilayer patches, facilitated sustained release of MC, providing antioxidant and antibacterial effects while maintaining mechanical stability and biocompatibility. Overall, MC demonstrates strong potential as a component of topical therapies and biomaterials for wound management [64,65,66,67,68].

#### 3.2.4. *Simmondsia chinensis* (Jojoba)

**Figure 7:**
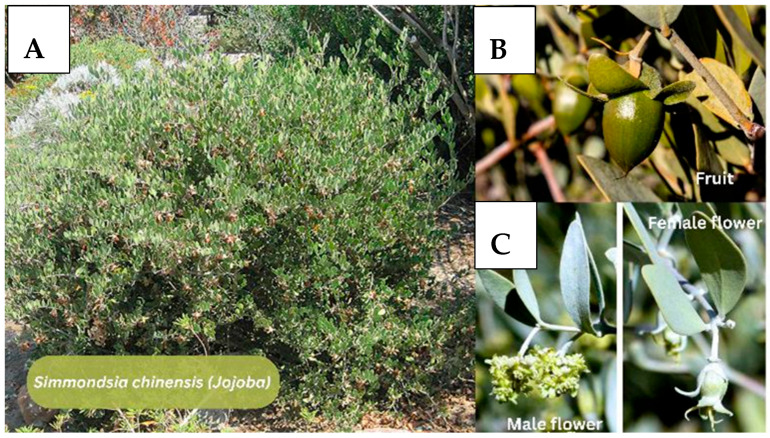
*Simmondsia chinensis* (Jojoba). (**A**) *S. chinensis* shrubs in their natural habitat. (**B**) Typical ovoid fruit. (**C**) Sexual dimorphism in flowers: clustered male flowers (left) and solitary female flowers (right), with the latter developing into fruit. Note. Figure created in Canva (Canva Visual Suite 2.0) using images from Adobe Stock. Photo rights © are the Author’s.

Multiple studies evaluated jojoba (Figure 7) liquid wax (JLW), topical jojoba wax, and methanolic jojoba seed extracts, demonstrating anti-inflammatory, extracellular matrix (ECM)-regenerative, and wound-healing properties across in vivo, ex vivo, and in vitro models.

In vivo anti-inflammatory effects of jojoba liquid wax (JLW) in rats and chick embryo models: JLW significantly reduced carrageenan-induced paw edema in rats and lowered prostaglandin E2 (PGE2) levels in inflammatory exudates. In the chick embryo chorioallantoic membrane (CAM) assay, JLW decreased granulation tissue formation, indicating suppression of angiogenesis and inflammation. In croton oil-induced ear edema, topical JLW reduced swelling, neutrophil infiltration (as measured by myeloperoxidase activity), and histopathological tissue damage. In an LPS-induced air pouch model, JLW decreased nitric oxide (NO) levels and TNF-α release, confirming its anti-inflammatory potential across multiple in vivo models [69].

Ex vivo anti-inflammatory and ECM-regenerative effects of topical jojoba wax on human skin: Topical application of jojoba wax to human skin organ cultures reduced LPS-induced IL-6, IL-8, and TNF-α secretion by ~30%, which is an effect similar to dexamethasone and enhanced by formulation with an emulsifier to improve bioavailability. The same application significantly increased mRNA and protein levels of pro-collagen III and enhanced hyaluronic acid synthesis, correlating with upregulation of TGF-β1. Preparations were well-tolerated, maintaining tissue integrity, and showing high cytocompatibility. Chemical analysis revealed consistent fatty acid and fatty alcohol profiles across industrial- and lab-scale batches, with eicosenoic acid as the major fatty acid, C_20:1_OH and C_22:1_OH as the primary fatty alcohols, variable tocopherol content, and stable phytosterol levels [28].

Wound-healing activity of methanolic jojoba seed extracts (He-Ne laser-treated): Methanolic extracts from 10 min He-Ne laser-treated jojoba seeds accelerated wound contraction and epithelialization in experimental models. By day 14, wound size reductions were 85.94% and 80.94% for two plant lines, surpassing control of jojoba extract (75.94%) and untreated groups (64.96%). The extracts increased angiogenesis markers including VEGF, TGF-β1, and HIF-1α, while reducing inflammatory mediators IL-1β, IL-6, TNF-α, and NF-κB, indicating coordinated enhancement of tissue repair alongside inflammation modulation [31].

In vitro wound-healing mechanisms of JLW in keratinocytes and fibroblasts: In HaCaT keratinocytes and human dermal fibroblasts, JLW exhibited very low cytotoxicity and accelerated scratch wound closure. Mechanistic analysis revealed that these effects are Ca(2+)-dependent and require activation of the PI3K-Akt-mTOR pathway and p38/ERK1/2 MAPKs. JLW stimulated collagen I synthesis in fibroblasts without affecting MMP-2 or MMP-9 gelatinase secretion, supporting its role in ECM regeneration and wound repair [70].

Across in vivo, ex vivo, and in vitro studies, jojoba preparations demonstrate multi-modal biological activity, including suppression of inflammatory mediators, promotion of extracellular matrix components (pro-collagen III, collagen I, hyaluronic acid), and enhanced wound repair via angiogenesis and cellular signaling pathways. These findings support the potential therapeutic and dermocosmetic application of jojoba for conditions involving inflammation, ECM degradation, and delayed wound healing. Further research is warranted to identify and characterize the active constituents responsible for these effects.

#### 3.2.5. *Opuntia phaeacantha* (*Chihuahuan prickly pear*)

**Figure 8:**
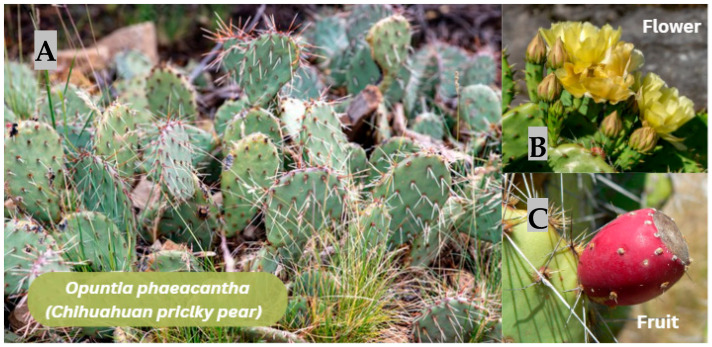
*Opuntia phaeacantha* (Chihuahuan prickly pear). (**A**) Cladodes with gray–green coloration, bearing long, light-colored spines (left panel). (**B**) Yellow flower (upper right panel). (**C**) Mature fruit (“tuna”) with reddish epidermis (lower right panel). Note. Figure created in Canva (Canva Visual Suite 2.0) using images from Adobe Stock. Photo rights © are the Author’s.

Hydrogels derived from the mucilage of *Opuntia ficus* (Figure 8) indica have exhibited key rheological properties that make them promising candidates for bioinks in 3D bioprinting and advanced biomedical systems. These hydrogels have been formulated with the incorporation of sucrose and calcium salts (chloride or carbonate) to optimize the formation of gel networks. They exhibit pseudoplastic (shear-thinning) behavior, which is essential for extrusion processes in bioprinters, where low-shear flow and structural stability after deposition are required.

The fit to the Casson model confirms their non-Newtonian nature, which is a desirable characteristic for bioinks that must maintain structural integrity post printing. In viscoelastic terms, hydrogels exhibit higher loss modulus (G”) values than storage modulus (G’), indicating predominantly viscous behavior, which favors adaptability and cohesion during printing. The negative ζ potential across the entire pH range suggests colloidal stability, although mucilage hydrogels show lower ionizability and phase stability than pectin-based systems, which could influence their interaction with cells and biomolecules. Multivariate photochemical analysis (PCA) and infrared spectroscopy reveal significant structural differences between mucilages and pectins, providing information on their functionality and potential modifications to improve biocompatibility. Taken together, these rheological and physicochemical properties position Opuntia mucilages as base materials for bioinks, with potential applications in tissue engineering, controlled drug delivery, and personalized biomedical systems [71].

#### 3.2.6. *Prospis grandulosa* (Mezquite)

**Figure 9:**
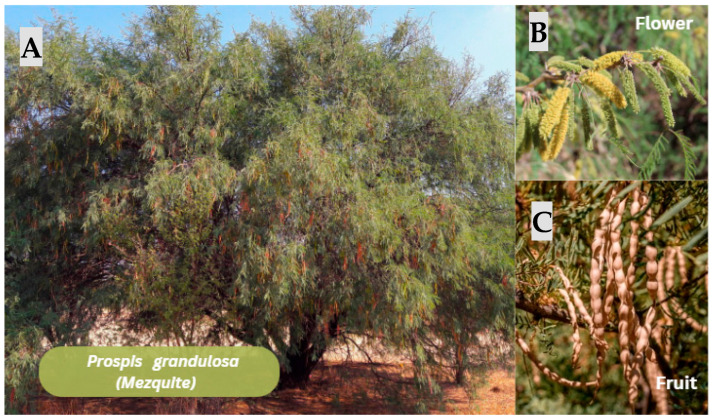
*Prosopis glandulosa* (Mesquite). (**A**) Entire tree showing a spreading canopy with slender, pinnate leaves (left panel). (**B**) Inflorescences consisting of cylindrical, yellow catkin-like flowers (upper right panel). (**C**) Mature fruits forming elongated, twisted pods characteristic of the species (lower right panel). Note: Figure created in Canva (Canva Visual Suite 2.0) using images from Adobe Stock. Photo rights © are the Author’s.

*Prosopis glandulosa* (Figure 9) provides two synergistic biomedical elements. Galactomannan biopolymer: suitable for hydrogels, films, and bioinks due to its pseudoplastic rheology, water retention, and compatibility with tissue-engineering polymers [70]. Bakuchiol phytochemical: clinically validated for collagen I/III stimulation, ECM remodeling, anti-inflammatory and anti-photoaging activity, and is ideal for dermal regeneration [72,73].

Evidence from other galactomannans demonstrates that these polysaccharides improve wound closure, fibroblast proliferation, collagen deposition, and ECM organization, supporting the projected biomedical use of *P. glandulosa* galactomannan. Existing evidence and the scientific gap regarding biomaterials derived from Prosopis glandulosa The current literature shows no substantial biomedical research has been performed using Prosopis glandulosa extracts directly as structural or functional components of biomaterials, including hydrogels, bioinks, or dermal regeneration matrices. However, phytochemical studies and cosmeceutical research consistently identify this species as a documented source of galactomannan and, more importantly, the meroterpenoid bakuchiol, which is a compound with well-established dermal bioactivity. Recent mechanistic and clinical studies demonstrate that bakuchiol can upregulate collagen types I and III, suppress MMP-1 and MMP-3, activate antioxidant pathways, and deliver retinol-like outcomes without irritation [73] This scientific gap highlights an unexplored opportunity: the leveraging of *P. glandulosa* metabolites and polysaccharides as promising candidates for advanced biomaterials targeting skin regeneration.

Summary of the cosmeceutical emulsions article and its relevance to dermal regeneration.

The article Preliminary Approaches to Cosmeceutical Emulsions Based on N-ProlylPalmitoyl Tripeptide-56 Acetate–Bakuchiol Complex demonstrates that bakuchiol-enriched emulsions show high physicochemical stability, strong antioxidant capacity, and excellent dermal tolerability, making them strong contenders for topical applications. Although the study does not examine Prosopis glandulosa directly, it acknowledges the species as a botanical source of the meroterpenoid, reinforcing its relevance within the natural production network of bakuchiol. The authors highlight the compound’s ability to stimulate regenerative signaling, enhance dermal firmness, and support extracellular matrix integrity, features that align closely with the functional requirements of bioactive hydrogels and 3D-printed scaffolds in skin tissue engineering [72].

Mechanistic evidence of bakuchiol in fibroblasts and its relevance for dermal ECM engineering:

Mechanistic studies demonstrate that bakuchiol—including the variant naturally present in *P. glandulosa*—enhances collagen I and III expression, reduces MMP-driven matrix degradation, mitigates oxidative stress, and promotes fibroblast cytoskeletal reorganization, ultimately improving dermal ECM architecture [73,74]. These molecular effects directly align with the performance goals of biomaterials designed to support ECM maturation, such as hydrogels, bioactive films, and 3D bioprinted constructs. Although the literature does not yet report the incorporation of *P. glandulosa*–derived bakuchiol into bio fabricated matrices, its biochemical profile and regulatory influence on ECM-related pathways provide a strong rationale for its exploration in next-generation dermal biomaterials.

Supported hypothesis on the influence of the meroterpenoid on the rheology of collagen–hyaluronic acid bioinks:

Considering its aromatic structure and moderate affinity for hydrophobic protein domains, bakuchiol may stabilize microdomains of the collagen triple helix, potentially increasing viscosity at low shear rates and improving filament stability during extrusion-based 3D printing. Its potent antioxidant activity could also protect collagen and hyaluronic acid from oxidative degradation, preserving molecular weight and reducing rheological drift during the printing process. In cell-laden bioinks, bakuchiol’s suppression of MMP activity and stimulation of ECM synthesis may promote a faster transition toward a structured viscoelastic network post printing, enhancing geometric fidelity and mechanical stability of printed constructs [73,75]. This framework represents a biomechanically grounded hypothesis that positions bakuchiol as a promising bioactive additive for collagen–HA bioinks.

#### 3.2.7. *Artemisa ludoviciana*

**Figure 10:**
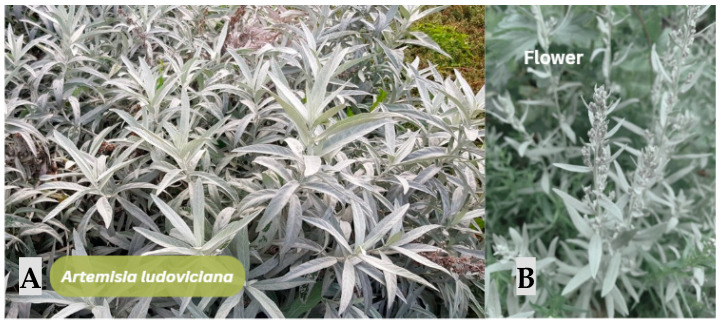
*Artemisa ludoviciana.* (**A**) Habit of the plant showing dense vegetative growth with the characteristic lanceolate, silvery-gray leaves typical of the species. (**B**) Close-up of the terminal flower structure. Note: Figure created in Canva (Canva Visual Suite 2.0) using images from Adobe Stock. Photo rights © are the Author’s.

In the current review, no studies were identified that employ *Artemisia ludoviciana* (Figure 10) directly in the fabrication of biomaterials, bioinks, or polymeric scaffolds for tissue engineering. Existing literature focuses exclusively on aqueous extracts, organic fractions, and essential oils evaluated for their pharmacological activities, revealing a conceptual and experimental gap regarding their integration into collagen- or hyaluronic acid-based bioinks. Nevertheless, the reported bioactivities of this species describe a pharmacological profile highly compatible with the biological and physicochemical requirements of advanced dermal biomaterials, thereby establishing a scientifically grounded foundation for future investigations.

The aqueous extract characterized by [76] demonstrates significant antinociceptive, anti-inflammatory, and antihyperalgesic activities in murine models. According to the analyzed document, the extract attenuates inflammatory responses in the carrageenan-induced edema model, reduces nociceptive behavior during the inflammatory phase of the formalin test, and improves thermal pain thresholds in hyper-glycemic mice, without inducing sedation or impairing locomotion. These effects are partially attributed to the sesquiterpene lactones achillin and dehydroleucodin, which modulate inflammatory mediators and pathways associated with COX-2 and NF-κB. Such mechanisms are particularly relevant for dermal biomaterials, as controlled inflammation facilitates fibroblast proliferation and orderly collagen deposition within 3D-printed environments.

Complementarily, the essential oil of A. ludoviciana evaluated by [77] exhibits marked antinociceptive activity associated with oxygenated monoterpenes such as 1,8-cineole, camphor, and borneol; these are metabolites with well-documented antimicrobial and antioxidant properties. From a biomaterials design perspective, these compounds represent promising candidates for incorporation into collagen or hyaluronic acid bioinks as biochemical modulators capable of reducing oxidative stress during extrusion, enhancing fibroblast protection, and imparting intrinsic anti-infective functionality to printed constructs. Taken together, these lines of evidence position A. ludoviciana as a floral resource with substantial potential for the development of bioactive biomaterials, provided that targeted studies on compatibility, stability, rheology, and cytocompatibility are conducted within the context of 3D bioprinting.

### 3.3. Projected Biofunctional Effects on Collagen Formation, Cell Proliferation, and ECM Remodeling from Studied Chihuahua Floral Sources

Although the mechanistic projections summarized in Table 2 stem from the studies previously examined and align with the biochemical processes described for skin wound repair, fibroblast behavior, and collagen stimulation it is essential to acknowledge that these interpretations remain reasoned hypotheses. They represent a logical extension of the available evidence and are intended to open the discussion rather than close to it. Still, when viewed collectively, the patterns that emerge offer a biologically meaningful direction for improving dermal bioinks.

What becomes clear is that several plant-derived metabolites including flavonoids, lignans, polysaccharides, and phenolic antioxidants consistently influence pathways that matter for printed dermal constructs: enhancing TGF-β/Smad activation, stabilizing the MMP/TIMP axis, limiting oxidative degradation of collagen, and promoting better organized fibrillar assembly within engineered scaffolds. These molecular behaviors are not abstract; they map directly onto the practical needs of next generation bioinks.

By incorporating these metabolites into gelatin, collagen, hyaluronic acid, or alginate-based formulations, we may create materials that respond more intelligently to the wound microenvironment supporting early fibroblast activation, moderating inflammatory peaks, and ultimately enabling a more functional and mechanically coherent neo dermis within 3D bioprinted constructs.

## 4. Discussion

### 4.1. Biofunctional Effects on Tissue Regeneration

In this sense, the projected biofunctional effects are in Table 2. act as more than a list of findings outlining a translational pathway. They connect phytochemical evidence with the rational, mechanism driven design of dermal bioinks capable of sustaining physiologically relevant skin regeneration in advanced 3D bioprinted models.

### 4.2. Biocompatibility of Natural Compounds Derived from Chihuahuan Flora

We view biocompatibility as the foundational checkpoint before integrating Chihuahua plant extracts or compounds into fibroblast oriented bioinks. Although each extract will require standardized assessments of cytotoxicity, reactivity, and immunological response, the broader biomedical literature already documents a long record of safe use for antioxidant metabolites such as NDGA, ellagic acid, Aloe-derived polysaccharides, and Opuntia mucilage. These compounds consistently support fibroblast viability, preserve morphology, and sustain proliferation even under pro-inflammatory stimuli Their antioxidant and microenvironment-stabilizing capacities also suggest a protective role during extrusion, crosslinking, and early post printing phases, where oxidative stress can compromise cell integrity. From our perspective, these trends reveal a favorable therapeutic window and justify systematic investigation of their incorporation into advanced bioink systems [49,51,52,58,59].

In vivo models reinforce this rationale. Hydrogels formulated with Larrea tridentata show smooth tissue integration without triggering chronic inflammation or fibrosis [50]. Similarly, Aloe and Jojoba derivatives enhance regenerative signaling upregulating TGF-Bβ1, collagen I/III and hyaluronic acid, while simultaneously reducing IL-6, IL-8, and TNF-α levels to maintain a balanced inflammatory profile that supports dermal repair [31,61,62]. Although most existing studies do not evaluate Chihuahuan flora specifically, they demonstrate that structurally related phytochemicals can modulate fibroblast behavior and extracellular matrix organization effectively. With this review, we aim to open a research pathway that encourages a detailed biocompatibility characterization of Chihuahuan plant compounds and evaluates their potential to enrich the biochemical and functional performance of next-generation dermal bioinks for skin tissue regeneration.

### 4.3. Bioethical Considerations and Sustainability Perspectives for the Use of Chihuahuan Flora Extracts in Biomaterial Development

We approach the ethical dimension of harvesting regional flora as a non-negotiable foundation for any future integration of Chihuahuan plant metabolites into biomaterial development. In alignment with the Secretariat of the Convention on Biological Diversity United Nations Environment Programme in 2011 and the *Nagoya Protocol on Access and Benefit-Sharing* [78,79] we consider *Larrea tridentata*, *Opuntia* spp., and other desert taxa as genetic resources that require transparent access frameworks, equitable benefit-sharing, and explicit respect for traditional knowledge. These international agreements guide our emphasis on traceability systems, community engagement, and ecological stewardship as mechanisms that reinforce scientific integrity. We also follow the ethical orientation established by UNESCO’s *Universal Declaration on Bioethics and Human Rights*, which links bioethics to the protection of biodiversity, the environment, and the rights of future generations [80]. Together, these frameworks justify our position that ethical governance is inseparable from the scientific exploration of regionally sourced phytochemicals.

From a sustainability perspective, we situate our evaluation within global principles for responsible use of biodiversity. The Addis Ababa Principles provide operational guidance for ensuring that biological resource utilization remains ecologically viable and socially equitable [81]. We also align with the United Nations Sustainable Development Goal 15, which promotes the conservation and sustainable use of terrestrial ecosystems and the prevention of biodiversity loss [82]. Desert plants inherently tolerate extreme climate conditions and minimal water availability, which reduces agricultural pressure relative to conventional crops and creates opportunities for low-impact biomass sourcing. We also envision extraction workflows evolving toward low-solvent, low-energy methodologies and the strategic use of plant residues such as cactus peel and Aloe by-products to obtain bioactive molecules without increasing harvesting pressure [83,84]. Although most antioxidant phytochemicals have been historically studied outside the context of Chihuahuan flora, their established biomedical relevance motivates the need to open a research pathway that evaluates these regional resources through rigorous biocompatibility, sustainability, and ethical criteria. This integrative framework positions Chihuahuan flora as potential contributors to next-generation dermal bioinks while remaining aligned with internationally accepted environmental and bioethical standards.

### 4.4. Enhancing Rheological Properties and 3D-Printing Performance

We treat rheological tuning as a central engineering lever for dermal bioinks. Hydrophilic polysaccharides with mucilage or pectin structures increase water molecules’ retention and promote shear-thinning and yield stress behavior that protects cells during extrusion and enhances print fidelity [85,86,87]. Recent work on polysaccharide bioinks which includes chitosan and pectin, alginate and pectin and pectin-based hydrogels; demonstrates controllable viscosity, rapid structural recovery, and high post printing viability, supporting their suitability as matrix enhancers for bioprinting applications [88,89,90,91]. Within this framework, we consider mucilage and pectin rich extracts from *Opuntia* and *Aloe* spp., together with flavonoids and lignans from *Larrea* and *Quercus* species, as promising natural modules for fine tuning viscosity, yield stress, and interlayer cohesion in collagen-based dermal bioinks [92].

Antioxidant phytochemicals provide a complementary layer of control. Polyphenol-based hydrogels are tannin, catechol, and ellagitannin and are included on crosslinked systems. Here, it enhances mechanical stability and provides ROS-scavenging activity, often with lower cytotoxicity compared to synthetic crosslinkers [93,94,95]. Their dual mechanical and biochemical function is highly relevant during photo crosslinking, where photo-initiators such as LAP or Irgacure generate ROS that can impair fibroblast phenotype and ECM production. An explicitly antioxidant bioink—rutin-modified methacrylate silk fibroin—demonstrated the reduction on intracellular ROS, improving chondrocyte viability, and enhanced extracellular matrix deposition after photocuring, providing direct experimental support for antioxidant reinforcement during bioprinting [96]. Likewise, a recent investigation of antioxidant loaded 3D-printed systems highlights the control on polyphenol release as a robust strategy to counteract oxidative stress in printed constructs [97]. When considered alongside broad evaluations of photo cross linkable biomaterials and photo-initiator-induced oxidative responses in bioprinting [98], these findings support the rationale that phenolic antioxidants from Chihuahuan flora can be incorporated into bioinks to enhance structural fidelity, mitigate ROS-mediated cellular damage, and stabilize fibroblast activity during printing and curing.

**Figure 11:**
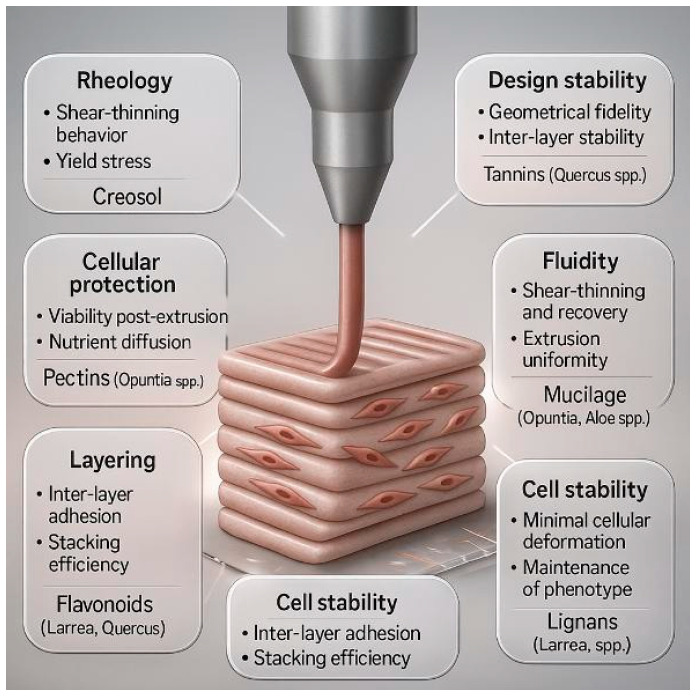
Proposed improvements in collagen 3D bioprinting using bioactive compounds from Chihuahua. Note: Image generated with Chat GPT-5.1 on 20 November 2025. The prompt used was as follows: “Create a high-resolution scientific illustration showing proposed improvements in collagen 3D bioprinting using bioactive compounds extracted from native plants of Chihuahua, Mexico. Include a 3D bioprinter depositing collagen-based bioink, molecular representations of bioactive compounds interacting with collagen fibers, and a schematic highlighting enhanced mechanical stability and cell viability. Use a clean, modern biomedical style with neutral laboratory colors and clear labeled components.”.

Figure 11 summarizes how some of these plant-derived metabolites like the mucilage and pectin’s derivatives from *Opuntia* and *Aloe*, flavonoids and lignans from *Larrea* and *Quercus*, and tannins from *Quercus* species map onto essential bioink properties including rheology, geometric fidelity, interlayer stability, and post extrusion cell protection. Collectively, these mechanistic routes offer a scientifically grounded pathway for engineering antioxidant enriched and collagen compatible dermal bioinks.

## 5. Conclusions

The comprehensive analysis of the Chihuahuan Desert flora presented in this review demonstrates that plant-derived metabolites—including nordihydroguaiaretic acid (NDGA) from *Larrea tridentata* [32,36], ellagic and gallic acids from *Prosopis* and *Quercus* species, flavonoids such as apigenin, luteolin, quercetin and naringenin from propolis and *Matricaria chamomilla* [26,29], and polysaccharides such as acemannan and mucilages from *Aloe vera* and *Opuntia* spp. [21]—possess significant and convergent biofunctional properties that are directly relevant to dermal regeneration and the engineering of next-generation bioactive bioinks.

Across the 39 studies evaluated, these compounds demonstrated the capacity to regulate key molecular cascades implicated in wound healing, including (i) upregulation of TGF-β1/Smad2/3 signaling, which promotes fibroblast activation, myofibroblast differentiation, and type I/III collagen transcription; (ii) activation of PI3K/Akt and ERK/MAPK pathways, supporting fibroblast proliferation and migration, as observed with *Allium cepa* extract [19] and jojoba-derived lipids [28]; and (iii) stimulation of pro-regenerative macrophage phenotypes (M2) via increased expression of ARG-1, IL-10, and TGF-β, particularly with *Aloe vera*-enriched hydrogels [25].

Simultaneously, potent antioxidant molecules such as NDGA, catechins, quercetin, luteolin, and gallic acid robustly activated Nrf2/ARE pathways, enhancing the expression of endogenous antioxidant enzymes (SOD, HO-1, catalase). This molecular protection reduces reactive oxygen species and prevents oxidative fragmentation of collagen fibrils, which is a key limiting factor in chronic or diabetic wounds [23]. In parallel, suppression of NF-κB and COX-2 signaling—consistently reported in chamomile extracts [27,30]—decreases the expression of matrix metalloproteinases (MMP-1, MMP-3, MMP-9), thereby restoring the MMP/TIMP balance required for orderly collagen deposition and ECM remodeling.

From the standpoint of biomaterials engineering, several of these phytochemicals also exhibit structural and rheological properties highly advantageous for bioink optimization. NDGA and ellagic acid can function as mild natural crosslinkers, enhancing print fidelity, shear recovery, and hydrogel cohesion without compromising cytocompatibility when used at controlled doses. Polysaccharides from *Aloe vera* and *Opuntia* improve viscoelasticity, shear-thinning behavior, and hydration dynamics, supporting stable extrusion and maintaining the geometric integrity of 3D-printed constructs. Their intrinsic antioxidant capacity may also protect cells embedded in photo-crosslinked bioinks from radical-induced cytotoxicity during curing.

Collectively, the evidence indicates that incorporating Chihuahuan plant metabolites into gelatin, collagen, hyaluronic acid, alginate, or GelMA-based bioinks may significantly enhance biological performance by promoting collagen synthesis, accelerating fibroblast proliferation, modulating inflammation, protecting ECM integrity, and improving dermal regeneration. These phytochemical-enriched bioinks represent a promising strategy to surpass the limitations of current inert or weakly bioactive formulations by providing biochemically intelligent materials that more closely replicate the molecular environment of native wound healing.

Nevertheless, future work must address key translational challenges, including standardizing extraction and purification protocols, defining dose–response effects to avoid toxicity (as NDGA may exhibit hepatotoxicity at high concentrations), conducting advanced rheological mapping of phytochemical–polymer interactions, and validating long-term safety and functional outcomes in large-animal wound models. Advancing these areas will be essential for regulatory and clinical translation.

In summary, this review establishes a strong scientific foundation for leveraging the unique phytochemical richness of the Chihuahuan Desert as a reservoir of bioactive molecules capable of enhancing collagen induction, ECM remodeling, and the functional performance of 3D-printed skin substitutes. Integrating these compounds into bioink engineering frameworks offers a transformative pathway toward bioactive, regenerative, and clinically relevant bioprinted dermal scaffolds.

## Figures and Tables

**Table 1:** Timeline: Recent phytochemical Mexican research, including bioactivity and applications in dermal scaffold research.

Vegetable Source	Highlighted Contributions	Active Compounds	Reference	Year
*Proposis glandulosa*	This study evaluated the wound-healing and anti-inflammatory activity of mesquite honey, obtained from *Prosopis glandulosa*, using a murine model. Eighteen male Wistar rats were used in the study. These rats received dorsal wounds through a standard 1 cm skin incision under aseptic conditions. Three treatments were administered to the rats: a control group, an experimental group (mesquite honey), and a reference drug group (1% silver sulfadiazine). The topical treatments were applied for 22 consecutive days. On days 1, 6, and 22 of treatment, the percentage of wound healing was calculated using digital photographs with ImageJ software version 1.48q, and histopathological parameters were evaluated using hematoxylin and eosin staining. The results showed that mesquite honey significantly improved wound healing and reduced inflammation compared to the control group and the reference drug group (*p* < 0.05).	Honey contains more than 200 bioactive components, including flavonoids, phenolic acids, enzymes, and antimicrobial peptides.	[18]	2025
*Allium cepa*	This study characterizes an extract of *Allium cepa*, including its terpenoid profile, and reports an antioxidant activity of 70%. In vitro wound-healing assays showed that a 15 mg/mL concentration significantly enhanced fibroblast proliferation and migration. These findings were further validated using O-carboxymethyl chitosan films containing 7% and 20% *w*/*w* extract, which exhibited higher cell viability after three days compared with control films. Overall, low extract concentrations improved cellular responses relevant to wound healing, supporting its potential as a bioactive component in polymeric biomaterials for skin regeneration.	Extract-characterization showing the presence of saponins, flavonoids.	[19]	2025
*Larrea tridentata*	This review assesses the antimicrobial and wound-healing potential of botanical extracts prepared using low- and high-ethanol concentrations. Wound types ranging from superficial abrasions to severe tissue damage were evaluated, including cases complicated by infection or systemic conditions. Extracts were tested against pathogens associated with human and canine wound infections (*Staphylococcus aureus*, *Pseudomonas aeruginosa*, *Staphylococcus pseudintermedius*, *Malassezia pachydermatis*). Human (HaCaT) and canine (CPEK) keratinocyte scratch assays showed that species such as Eucalyptus globulus, Juglans nigra, *Larrea tridentata*, Salvia officinalis, Zingiber officinale display broad antimicrobial activity, while Achillea millefolium, Aloe vera, and Usnea barbata improved wound closure. These findings support their potential incorporation into new therapeutic formulations.	Plant extracts	[20]	2025
*Aloe vera*	This work investigates the integration of *Aloe vera* extract into collagen–polyurethane hydrogels to develop multifunctional wound dressings. *Aloe vera* provides healing, anti-inflammatory, moisturizing, and antimicrobial benefits due to its polysaccharides, proteins, vitamins, and anthraquinones (e.g., aloin, emodin). Hydrogels containing 20–60 wt% extract formed semi-interpenetrating polymer networks (semi-IPNs), with higher extract content increasing crosslinking (38 ± 3%) and superabsorbent capacity (2850 ± 210%). The 60 wt% formulation achieved optimal viscosity, biodegradation resistance, antibacterial effects (E. coli 78%; S. aureus 57%), and controlled ketorolac release (65% at pH 5.5). Biocompatibility assays confirmed fibroblast and monocyte proliferation, non-hemolytic behavior, and increased TGF-β1 secretion. These results highlight the potential of Aloe vera-loaded hydrogels as multifunctional wound-healing systems.	Phenolic compounds	[21]	2025
*Opuntia* ssp.	The study evaluated the in vitro biological activities of polysaccharides obtained from *Opuntia pulp* (POS) and their efficacy as a wound-healing agent in a diabetic rat model. The methodology included the evaluation of antioxidant properties, the study of POS inhibition of β-amylase and acetylcholinesterase, as well as the anti-inflammatory and antihemolytic effects of POS. The results showed that POS possesses antioxidant activity, effectively neutralizing nitric oxide radicals and thus protecting DNA from damage. Furthermore, POS showed potent inhibition of β-amylase with an IC50 value of 1968 µg/mL and of acetylcholinesterase with an IC50 value of 157.33 µg/mL. Furthermore, the results indicated that POS exhibited potential anti-inflammatory activity, preserving erythrocyte membrane integrity, and inhibiting hemolysis. The use of POS in a diabetic wound model showed a substantial improvement in healing and accelerated wound closure after 15 days of induction.	Polysaccharides	[22]	2025
*Chihuahua propolis*	This study evaluated the wound-healing effects of an ethanolic extract of *Chihuahua propolis* in diabetic mice. Chemical analysis and in vivo assays demonstrated significantly improved wound closure in full-thickness wounds, suggesting that the extract mitigates diabetes-induced impairments in the healing process.	Nine phenolic and flavonoid compounds were identified by HPCL-DAD: Catecol, Catequin, Naringenin, Naringin, Genistein, Lutenoin, Apigenin, Chrysin, Bicalein.	[23]	2024
*Artemisia ludoviciana*	This study proposes the use of plant extracts for phytotherapeutic purposes in the treatment of dermatophytosis. *Microsporum canis* is one of the most frequent causes of dermatophytosis in humans, which motivated the attempt to apply extracts obtained by Soxhlet extraction from *Artemisia ludoviciana* and *Cordia boissieri*, which were characterized. The extracts were evaluated against a commercial strain of *M. canis* (ATCC-11621) using the method described in the Clinical and Laboratory Standards Institute protocol M38-A to obtain the minimum inhibitory concentration (MIC) and the minimum fungicidal concentration (MFC). These concentrations were tested in a human keratinocyte cell line. The results showed that the extracts of *Artemisia ludoviciana* and *C. boissieri* exhibited MIC values of 2500 and 1250 µg/mL, and MFC values of 5000 and 2500 µg/mL against the study strain, respectively.	Sterols and triterpenes, Sesquiterpene lactones, Coumarins, Saponins, Flavonoids, Aromatics, Phenolic oxides	[24]	2024
*Aloe vera*	This work presents the development of an injectable hydrogel (PDMA-GelMA-AV) incorporating *Aloe vera*, gelatin methacrylate (GelMA), and polydopamine methacrylamide (PDMA) to overcome limitations of natural hydrogels, such as weak mechanical strength and low adhesiveness. The hydrogel demonstrated controlled degradation, sustained release of *Aloe vera* bioactives, enhanced fibroblast proliferation and migration, reduced pro-inflammatory mediators (TNF-α, IL-1β, iNOS), and increased TGF-β and ARG expression. In vivo studies confirmed accelerated wound closure and biocompatibility.	Phenolic compounds	[25]	2024
*Matricaria chamomilla* L.	This review synthesizes current evidence on *chamomile*’s traditional uses and its molecularly supported therapeutic potential in inflammatory skin diseases. *Chamomile* contains over 120 secondary metabolites, including flavonoids, terpenoids, sesquiterpenes, essential oils, and organic acids. These constituents contribute to antioxidants, anti-inflammatory, antibacterial, antispasmodic, sedative, hepatoprotective, neuroprotective, and antitumor activities. Evidence supports its efficacy in dermatological applications through synergistic interactions among its phytochemicals.	Flavonoids, flavones apigenin, luteolin, patuletin, and their glucosides (apigenin 7-O-β-D-glucoside, luteolin 7-O-β-D-glucoside, luteolin 4′-β-glucoside, luteolin 6-hydroxy-7-glucoside) quercetin and its glucosides, including quercetin 3-glucoside and rutin; the flavanone naringenin; and the monoterpenes geraniol, bornanol, citronellol, and menthol.	[26]	2024
*Matricaria chamomilla* L.	In this study, the authors developed a dressing containing a hydrogel and a fibrous structure with multifunctional characteristics that enhances the efficacy of skin healing. A hydrogel made with sodium alginate (SA)/gelatin (Gel) was enriched with *Matricaria chamomilla* L. extract and silver sulfadiazine (SDA) as antibacterial agents, crosslinked with genipin and calcium chloride. The hydrogel was coated with electrospun polyacrylonitrile (PAN) nanofibers to fabricate the bilayer dressing. FESEM images showed that the PAN nanofibers had a continuous, smooth morphology and were free of microspheres, demonstrating compatibility between the hydrogel and the fibers. The prepared material exhibited mechanical properties such as an elastic modulus (2.4 ± 0.2 MPa), tensile strength (6.2 ± 0.5 MPa), and elongation at break (21.8 ± 1%). The material exhibited adequate biodegradability, cytocompatibility, and antibacterial performance against both Gram-positive and Gram-negative strains. The silver sulfadiazine release profile was developed via a Fick diffusion mechanism, ensuring sustained release. In vivo tests demonstrated that dressing promoted wound closure, re-epithelialization, and collagen deposition.	Flavonoids, flavones apigenin, luteolin, patuletin, and their glucosides (apigenin 7-O-β-D-glucoside, luteolin 7-O-β-D-glucoside, luteolin 4′-β-glucoside, luteolin 6-hydroxy-7-glucoside) quercetin and its glucosides, including quercetin 3-glucoside and rutin; the flavanone naringenin; and the monoterpenes geraniol, bornanol, citronellol, and menthol.	[27]	2024
*Simmondsia chinensis*	The study evaluated the anti-inflammatory activity of jojoba wax and its impact on the production of extracellular components through topical application. The results showed the fatty acid and fatty alcohol profiles of two industrially cold-pressed jojoba waxes and two laboratory-scale jojoba waxes, as well as their tocopherol and phytosterol content. The study also examined the ability of jojoba wax to reduce the amount of pro-inflammatory cytokines. Furthermore, the effect of jojoba wax on pro-collagen and hyaluronic acid synthesis was studied. The results showed that topically applied jojoba wax reduced the secretion of IL-6, IL-8, and LPS-induced TNFα by approximately 30% compared to untreated skin. Additionally, the treatment increased mRNA levels, collagen III protein, and hyaluronic acid synthesis.	Tocopherols, phytosterols, eicosenoic acid was the primary fatty acid, and fatty alcohols were equally distributed between C_20:1_OH and C_22:1_OH.	[28]	2024
*Chihuahua propolis*	The study demonstrated that 10% *w*/*v* Mexican propolis (ChEEP) exhibits no acute toxicity and possesses antibacterial activity against Gram-positive bacteria such as Staphylococcus aureus and Staphylococcus epidermidis. Furthermore, it showed an anti-inflammatory effect. After 14 days of topical treatment, greater wound contraction, reduced healing time, and increased tensile strength were observed, along with the formation of type I collagen at the injury site.	Eight flavonoid compound where identified in propolis extract: Naringin, Naringenin, aempferol, quercetin, Acacetin, Luteolin, Pinocembrin, Chrysin.	[29]	2023
*Matricaria chamomilla* L.	The authors prepared environmentally friendly and biodegradable nanofibers (NFs) based on N-(3-sulfopropyl) chitosan/poly(ε-caprolactone) incorporating zeolite imidazolate-8 nanoparticles (ZIF-8 NPs) and chamomile essential oil (MCEO) using electrospinning for their effectiveness as wound dressing scaffolds. The fabricated material was characterized morphologically, hydrophilically, and thermally. Scanning electron microscopy (SEM) analyses showed that the addition of ZIF-8/AEM NPs (PCL/SPCS (90:10) with a diameter of 90 ± 32 nm) affected the fiber diameter. The material with uniform ZIF-8/PCL/SPCS and loaded with chamomile essential oil (MCEO) exhibited improved cytocompatibility, proliferation, thermal stability, and mechanical properties compared to the pure nanofibers. The results also demonstrated that the formulated nanofibers exhibited promising adhesion and proliferation against human foreskin fibroblasts-2 (HFF-2 cell line). The prepared nanofibers showed excellent antibacterial activity against Staphylococcus aureus and Escherichia coli, with inhibition of 32.3 µm and 31.2 µm, respectively.	Flavonoids, flavones apigenin, luteolin, patuletin, and their glucosides (apigenin 7-O-β-D-glucoside, luteolin 7-O-β-D-glucoside, luteolin 4′-β-glucoside, luteolin 6-hydroxy-7-glucoside) quercetin and its glucosides, including quercetin 3-glucoside and rutin; the flavanone naringenin; and the monoterpenes geraniol, bornanol, citronellol, and menthol.	[30]	2023
*Simmondsia chinensis*	This research evaluated the stimulatory effects of He-Ne laser irradiation on the bioactivity of phytochemicals in jojoba plants. Jojoba seeds were irradiated for 5, 10, and 15 min prior to in vitro germination. Additionally, a comparative study of wound healing and antimicrobial activity was conducted using methanolic extracts of non-irradiated (control) and 10 min irradiated seeds, employing an excision wound model in male Wistar rats and an inhibition zone assay. The results showed that, when comparing the control plant extracts and the 10 min treatments, the latter exhibited higher wound contraction percentages and shorter epithelialization periods.	N-Methyl-L-proline, 1-[4-hydroxy-3-methylphenyl]ethanone, Pyrrolidine, Methyl alpha-D-glucopyranoside, Levoglucosan, D-Glucose, Methyl palmitate, l-[+]-Ascorbic acid 2,6-dihexadecanoate, D-Fructose 3-O-methyl, Arbutin, Acetosyringone, [4-hydroxyphenyl] acetonitrile, L-Thymidine. Undercane, Propanoic acid, Phytol.	[31]	2023
*Larrea tridentata*	This review aims to summarize recent findings on the phytochemical composition and pharmacological potential of *Larrea tridentata*. Phytotherapy has been the primary approach to treating infectious and non-infectious diseases for centuries. Although still practiced with remarkable success in many regions, it is often underestimated due to its limited empirical basis compared to Western medicine. *Larrea tridentata*, a perennial shrub native to Northern Mexico and the southwestern United States, has long been used in traditional medicine for conditions such as infertility, rheumatism, arthritis, colds, diarrhea, skin disorders, pain, inflammation, and obesity. Scientific studies have confirmed its broad-spectrum antioxidants, antitumor, neuroprotective, regenerative, and antimicrobial properties, although some compounds exhibit hepatotoxicity and nephrotoxicity.	ellagic acid, gallic acid, catechins, methyl gallate, cinnamic acid resorcinol, kaempferol, quercetin, nordihydroguaiaretic acid (NDGA), thymol, carvacrol,3-[(O-(4-O-sulfo-b-d-glucopyranosyl)-(1→3)-a-L-arabinopyranosyl) oxy]olean-12-en-28-oic acid b-d-glucopyranosyl ester sodium salt.	[32]	2022
*Artemisia ludoviciana*	This research focused on evaluating records showing the use of *Artemisia ludoviciana* for the treatment of tuberculosis. Therefore, the combination of antibiotics and plant extracts could represent an attractive alternative as a novel antimycobacterial agent. The alcoholic extract of *A. ludoviciana* showed an MIC of 250 μg/mL against a clinical strain of *M. tuberculosis*. In the case of ex vivo cytotoxicity of the extract applied to the THP-1 cell line, it showed an IC50 of 20 μg/mL. Additionally, the toxicity test of the Artemia model showed moderate toxicity when the *A. ludoviciana* extract was administered, with an LC50 of 195.64 μg/mL. Finally, the inflammatory response of THP-1 cells exposed to the extract did not show an increase in the secretion of interleukin-6 and -10.	Achillin as the major component, meanwhile the minor components such as thujone and stigmasterol.	[33]	2022
*Prosopis glandulosa*	This study aimed to develop and characterize nanofibers *incorporating Prosopis glandulosa (mesquite gum, MG)* combined with biodegradable polymers (pullulan and chitosan) using the Forcespinning^®^ technique with MG concentrations of 18.1% and 28% by weight, combined with pullulan and chitosan. Furthermore, antibacterial activity against Escherichia coli (Gram-negative) and Bacillus megaterium (Gram-positive) was tested using disk diffusion. The results showed that the nanofibers were continuous and long, with their diameter increasing with increasing MG concentration (523 ± 180 nm for 18.1 wt% and 760 ± 225 nm for 28 wt%). Infrared spectra confirmed the presence of MG functional groups (carboxylic acids, amides, polysaccharides), indicating successful incorporation without degradation. Furthermore, increasing MG concentrations enhanced thermal stability, and the crosslinked fibers exhibited hydrophilic behavior with 3 to 6 percent water absorption. Regarding the wound-healing potential of the nanofibers, Pulluan with MG-28 nanofibers showed inhibition zones of approximately 11 mm against E. coli and approximately 10 mm against B. megaterium. Furthermore, the MG solution inhibited bacterial growth, suggesting that tannins alter bacterial membranes and metabolic activity; the latest results imply that nanofibers with MG show a promising bioactive wound dressing material.	Polysaccharides (D-galactose, L-arabinose, D-mannose), antioxidants, alkaloids, flavonoids, and tannins.	[34]	2022
*Mimosa tenuiflora*	This work explored the environmentally friendly synthesis of silver nanoparticles (AgMt NPs) using a *Mimosa tenuiflora* extract (MtE) as a reducing agent. The nanoparticles, with an average size of 21 nm and an fcc crystal structure, were characterized by UV-Vis spectroscopy, XRD, HRTEM, XPS, TGA, and antioxidant assays (DPPH, total polyphenols). Residual MtE was detected in the AgMt NPs even after purification. Subsequently, carbopol-based hydrogels incorporating either MtE or AgMt NPs (MtE-G and AgMt NP-G) at 100 µg/g were prepared and analyzed by UV-Vis, IR, and rheology. Antimicrobial activity against Staphylococcus aureus and Escherichia coli was tested, while burn healing was evaluated in Wistar rats using histopathological analysis. Compared to commercial Ag NP gels, AgMt NP-Gs demonstrated superior bactericidal and anti-inflammatory effects, promoting faster wound recovery and positioning them as a promising strategy for burn treatment.	Plant extract	[35]	2021
*Larrea tridentata*	This work presents a study on *Larrea tridentata (creosote bush)*, a perennial shrub native to the Chihuahuan, Sonoran, and Mojave deserts, recognized for its diverse secondary metabolites, particularly lignans such as nordihydroguaiaretic acid (NDGA). For over seventy years, this species has been investigated for its broad biological activities, including bactericidal, fungicidal, nematicidal, protozoal, and antiviral effects. Historically, NDGA has been used as an antioxidant in food preservation, and recent studies highlight its potential anticancer properties. Despite extensive pharmacological research, information on its application in livestock production remains limited, with only a few studies in sheep and poultry. Preliminary findings suggest that creosote bush may improve productive performance and modulate the gut microbiota, indicating promising prospects for animal health and nutrition.	Phenolics lignans, nordihydroguaiaretic acid.	[36]	2021
*Opuntia* ssp.	This study assessed the wound healing activity of Opuntia ficus indica seed oil (OFI) and its self-nanoemulsifying drug delivery system (OFI-SNEDDS) formula in a rat model of full-thickness skin excision. The wound healing activity of OFI and OFI-SNEDDS was studied in vivo. The last formulation had a droplet size of 50.02 nm, also the formula showed best healing activities as compared to regular Opuntia seed oil-treated rats on day 14 of wounding. The histopathological examinations confirmed the healing effect. The self-nanoemulsifying system exhibited greater antioxidant and anti-inflammatory activities as contrasted to Opuntia seed oil-treated animals. Both systems increase significantly enhanced hydroxyproline skin content and upregulated Col1A1 mRNA expression, accompanied by enhanced expression of transforming factor-beta (TGF-β). Finally, OFI-SNEDDS increases angiogenesis as shown by the rise in vascular endothelial growth factor (VEGF).	Palmitic acid (10.68%), linoleic acid (5.9%), oleic acid (8.16%), campesterol (6.58%) and β-sitosterol.	[37]	2021
*Mimosa tenuiflora*	This study explored the incorporation of a *Mimosa tenuiflora* extract into O-carboxymethyl chitosan films and its subsequent characterization. The extract was obtained from the bark of the plant specimen by aqueous extraction, followed by ethanol precipitation, filtration, and concentration. Its optimum concentration was determined using a scratch wound assay. The mechanical properties, chemical composition, and structural characteristics of the composite films were analyzed. Antimicrobial performance against two bacterial strains was evaluated by turbidimetry, and enzymatic degradation was assessed using lysozyme assay. Biocompatibility and cytotoxicity were evaluated in fibroblast cultures, complemented by in vivo wound healing studies in mice. The results demonstrate that the addition of *Mimosa tenuiflora* extract promotes fibroblast proliferation and accelerates wound closure, highlighting its potential as a novel biomaterial for skin regeneration.	Plant extract	[38]	2020
*Aloe vera*	This study presents the development of natural fibers composed of zein, polycaprolactone (PCL), and collagen, manufactured and functionalized with zinc oxide nanoparticles (ZnO NP) and *Aloe vera* extract (NF/ZnO/Alv). Morphological, mechanical, thermal, and wettability analyses revealed that fibers with a zein/PCL ratio of 70:30, ZnO (1 wt.), and *Aloe vera* (8 wt.) exhibited optimal stability and strength. Hydrophilicity improved with lower zein/PCL ratios. Cytocompatibility was confirmed by fibroblast adhesion assays at 24 and 72 h. Antimicrobial tests demonstrated significant inhibition against Staphylococcus aureus (19.23 ± 1.35 mm) and Escherichia coli (15.38 ± 1.12 mm). These findings suggest that NFs/ZnO/Alv compounds offer a multifunctional platform for wound healing, combining structural support, antimicrobial activity, and improved cell adhesion.	Phenolic compounds	[39]	2020
*Caryaillinoinensis*	A systematic review showed that *pecan nuts*, oils, and by-products reduce cardiovascular disease risk and have notable anti-inflammatory effects, associated with their rich lipid and polyphenolic composition.	Phytosterols, phospholipids, sphingolipids, squalene, polyphenols and low amounts of carotenoids and tocotrienols.	[40]	2018

**Table 2:** Projected biofunctional effects on collagen formation, cell proliferation, and ECM remodeling from previous Chihuahuan floral sources.

Floral/Plant Resource	Verified Effects on Collagen/ECM (from Reviewed Studies)	Biomaterial Context	Three-Dimensional Bioink-Relevant Projection
*Allium cepa*	*Allium cepa* extract in O-carboxymethyl chitosan films increases fibroblast proliferation and migration, accelerates in vitro scratch closure, and shows high cell viability at low doses.	O-carboxymethyl chitosan (OCMC) bioactive films incorporating *A. cepa* extract.	OCMC–*Allium cepa* systems already function as bioactive polysaccharide scaffolds; therefore, they could be adapted into printable chitosan-based bioinks, where low extract concentrations provide pro healing and antioxidant cues without compromising rheology.
*Aloe vera*	Collagen/polyurethane hydrogels loaded with aloe enhance wound healing, promote ECM deposition, support re-epithelialization, and improve dermal structural quality; Aloe polysaccharides contribute strong hydration and regenerative effects.	Collage/polyurethane hydrogels and various Aloe-based hydrogels for wound dressings.	Aloe polysaccharides (especially acemannan) already operate inside hydrated networks and modulate moisture and healing, suggesting high potential as viscosity modifiers, hydration stabilizers, and cell-protective additives in collagen/GelMA/HA-based bioinks.
*Carya illinoinensis* (*pecan*)	Pecan kernels rich in phenolic compounds show strong antioxidants and metabolic regulatory activity; evidence relates to systemic oxidative balance rather than local dermal collagen production.	Not used as wound biomaterials; primarily evaluated as a nutritional antioxidant source.	Direct relevance to dermal bioinks is low; polyphenolic fractions *could* hypothetically serve as antioxidant additives, but no skin or 3D-printing models exist, making this projection speculative.
*Chihuahua propolis*	Ethanolic propolis extracts improve wound closure in murine models, including diabetic wounds; reduce inflammation; and promote stronger collagen fiber organization. Topical 10% formulations show antibacterial activity and no acute toxicity.	Topical gels and ointments formulated with ethanolic Chihuahua propolis.	Propolis flavonoids already act within semi solid matrices and provide potent antioxidant/anti-inflammatory effects; thus, they can be incorporated into bioinks via nano or micro encapsulation, enhancing redox stability without disturbing polymer crosslinking.
*Larrea tridentata*	Hydroalcoholic extracts exhibit broad antimicrobial activity with significant antioxidant capacity and improve scratch closure dynamics in vitro. Polyphenols and lignans modulate oxidative and microbial loads relevant to wound repair.	Liquid extracts were tested for antimicrobial and antioxidant properties.	Due to strong redox and antimicrobial effects, *L. tridentata* could serve as a controlled dose bioactive additive in bioinks, improving microenvironmental stability. However, cytotoxicity dose requires strict concentration control. No 3D-printed models yet exist.
*Simmondsia chinensis* (*jojoba*)	Jojoba wax increases pro-collagen III and hyaluronic acid synthesis in human ex vivo skin models; reduces inflammatory mediators; and laser-enhanced extracts exhibit increased antimicrobial and wound-healing activity.	Topical formulations (wax/oil) and methanolic extracts; some studies include He–Ne laser enhancement.	Because jojoba’s main components are lipids, its integration into aqueous polymeric networks requires stabilization, but nanoemulsified jojoba fractions could act as bioactive inclusions in HA- or collagen-rich bioinks, enhancing hydration and ECM-upregulation cues if properly emulsified.
*Opuntia* spp. (*pulp polysaccharides and seed oil*)	Opuntia pulp polysaccharides showed antioxidant, anti-inflammatory and antihemolytic effects and significantly improved healing and wound closure in a diabetic rat model. Opuntia ficus indica seed oil and its SNEDDS significantly increased hydroxyproline content, upregulated Col1A1 and TGF-β expression, and boosted VEGF-mediated angiogenesis in skin, directly supporting collagen deposition and ECM maturation.	POS tested as a solution in diabetic wounds (no scaffold); OFI seed oil delivered either as oil or self-nanoemulsifying system in full-thickness excision model.	Opuntia polysaccharides can function as hydrating, shear-thinning polysaccharide components in bioinks, providing antioxidant protection and improved healing in diabetic microenvironments. OFI-SNEDDS data supports its use as a pro-collagen, pro angiogenic nano-additive to enrich dermal bioinks with lipids and sterols that upregulate Col1A1/TGF-β, potentially improving ECM quality in printed grafts.
*Artemisia ludoviciana*	Extracts showed fungistatic and fungicidal activity against *Microsporum canis*, with defined MIC/MFC, and moderate toxicity in cell and Artemia models. Separate work showed antimycobacterial activity against *M. tuberculosis* without elevating IL-6 or IL-10. There is no direct evidence of increased dermal collagen, but clear anti-infective and controlled inflammatory profiles that may indirectly support ECM preservation.	Evaluated as alcoholic extracts; no wound scaffold integration in the cited studies.	Artemisia ludoviciana appears most suitable as a targeted antifungal/antimycobacterial additive for bioinks intended for infected or high-risk wounds. Any integration into 3D bioinks should focus on localized, low dose delivery (e.g., encapsulation in microparticles) to exploit its antimicrobial action while minimizing cytotoxicity and avoiding disruption of fibroblast-driven ECM deposition.
*Matricaria chamomilla*	Reviews report strong antioxidant, anti-inflammatory and dermatological benefits driven by flavonoids (apigenin, luteolin, quercetin derivatives) and terpenoids ([26]). In bilayer SA/Gel hydrogels enriched with chamomile extract and silver sulfadiazine, in vivo tests showed improved wound closure, re-epithelialization and collagen deposition. Chitosan/PCL nanofibers with chamomile improved fibroblast proliferation and showed potent antibacterial activity.	(i) SA/gelatin hydrogels crosslinked with genipin/CaCl_2_ plus chamomile extract and silver sulfadiazine, coated with PAN nanofibers; (ii) chitosan/PCL nanofibers with ZIF-8 NPs and chamomile essential oil; (iii) extensive pharmacological review data.	Chamomile extracts and essential oil are promising soothing, antioxidant, and collagen-supportive additives for dermal bioinks. Their integration into alginate/gelatin or chitosan/PCL-based constructs could improve cytocompatibility, antibacterial protection and collage-rich ECM remodeling, while ZIF-8-like carriers suggest strategies for controlled release of chamomile phytochemicals in printed grafts.
*Mimosa tenuiflora*	(a) O-carboxymethyl chitosan films containing *M. tenuiflora* accelerate fibroblast proliferation and wound closure, with good biocompatibility and antimicrobial performance. (b) Silver nanoparticles synthesized with *Mimosa* extract (AgMt NPs-G) show potent bactericidal and anti-inflammatory effects and improve second-degree burn healing.	(a) OCMC/Mimosa composite films. (b) Hydrogels incorporating AgNPs produced via green synthesis using *Mimosa* extract.	Since *Mimosa tenuiflora* already operates effectively within chitosan and hydrogel matrices, it provides a near to direct translation path to bioinks: OCMC offers suitable viscosity/printability, while Mimosa polyphenols add antimicrobial and regenerative bioactivity. Ag-NP systems may inspire antimicrobial bioinks, with careful cytotoxicity monitoring.

## Data Availability

No new data were created or analyzed in this study. Data sharing is not applicable to this article.
